# Unusual Presentation of Gallbladder Fossa Abscess Following Open Cholecystectomy in a Patient With Cholecystitis: A Case Report

**DOI:** 10.7759/cureus.25274

**Published:** 2022-05-24

**Authors:** Hirva Vyas, Ethan Burg, Ramtin Moradi, Alan Chu, Davood K Hosseini

**Affiliations:** 1 Internal Medicine, Hackensack University Medical Center, Hackensack, USA; 2 Medicine, Hackensack Meridian School of Medicine, Hackensack, USA; 3 Internal Medicine, Richmond University Medical Center, Staten Island, USA

**Keywords:** chronic cholecystitis, ct scan, cholecystitis, cholecystectomy, gallbladder fossa abscess

## Abstract

Cholecystectomy is one of the most commonly performed surgical interventions, and laparoscopic cholecystectomy is the standard intervention with open cholecystectomies having declined nowadays. Similar to other surgical procedures, cholecystectomy carries its own risks including sepsis, bleeding, damage to surrounding tissues, bile leakage, and abscess formation. Abscess formation can be due to a variety of reasons such as infection or gallstone spillage during surgery with the latter being more common to laparoscopic surgery. Here we describe a patient with an unusual presentation of gallbladder fossa abscess following open cholecystectomy.

## Introduction

Acute cholecystitis is the inflammation of the gallbladder, which occurs due to obstruction of the cystic duct, resulting in bile stasis, and subsequently inflammation and edema of the gallbladder wall. Cholelithiasis is the most common cause of cystic duct obstruction, and accounts for 95% of acute cholecystitis [1].

Laparoscopic cholecystectomy is the standard approach for this procedure with open cholecystectomies having declined nowadays [2-6]. This is in large part due to laparoscopic cholecystectomies having better patient outcomes with reduced morbidity, mortality, infection, and shorter hospitalizations [3]. However, like any surgical procedure, cholecystectomy carries its own risks such as sepsis, bleeding, damage to surrounding tissues, bile leakage, and abscess formation [2-4, 6, 7]. Abscess formation as seen in this case can be due to a variety of reasons such as infection or gallstone spillage during surgery with the latter being more common to laparoscopic surgery [4,6,7].

## Case presentation

**Figure 1:**
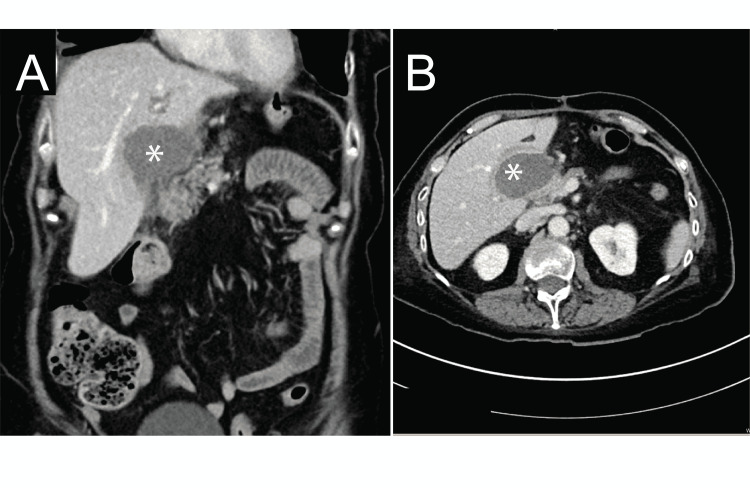
Abdominal computed tomographic (CT) scan. Abscess with complex fluid collection measured 5.3 x 3.6 x 5.8 cm^3^ (Asterix) in coronal (A), and axial view (B).

A 71-year-old female with a history significant for Barrett's esophagus with distal esophageal adenocarcinoma status post neoadjuvant chemotherapy, radiation therapy and Ivor Lewis Esophagectomy and following open cholecystectomy secondary to chronic cholecystitis, presented eight months later with right upper quadrant abdominal pain. Right upper quadrant abdominal pain was ongoing for a month, and it was accompanied by few days of non-bilious vomiting and fevers. On admission, she was found to be afebrile with a blood pressure of 130/90, heart rate of 85, and oxygen saturation of 97%. Laboratory findings were remarkable for leukocytosis with neutrophilic predominance (87.9%) and a potassium of 3.3. Computed tomography (CT) scan of the abdomen revealed a new 5.3 x 3.6 x 5.8 cm^3^ fluid collection (Figure 1).

The patient underwent a Jackson-Pratt drain placement and subsequent body fluid cultures showed Streptococcus intermedius and blood cultures grew the same organism. She was initially treated with aztreonam and flagyl until sensitivities resulted and afterwards, she received a course of vancomycin with resolution of her symptoms (as the patient was allergic to cephalosporins).

## Discussion

Cholecystectomy is one of the most commonly performed surgical interventions with an estimated 300,000 cases performed in the United States each year due to gallstones [2].

Adverse events such as abscess formation can lead to a post-cholecystectomy syndrome which most commonly presents with abdominal pain, dyspepsia, fever, and jaundice, however, it can present with other symptoms such as vomiting, pseudocysts, and liver abscess [4]. Abscesses usually present within a couple of weeks of cholecystectomy [8,9]. In the event of a gallstone spillage or retained stone, complications such as intra-abdominal abscess can manifest years following the initial surgery. These abscesses presenting years after cholecystectomy are commonly colonized by E. coli and have a female predominance. Despite prior occurrences, abscess formation protracted from surgery constitutes a rare occurrence [6,7].

## Conclusions

The case described here is an unusual presentation of a gallbladder fossa abscess, which was diagnosed eight months following an open cholecystectomy. Previous studies revealed that gallbladder fossa abscess may have been a potential distant complication of a spilled or retained gallstone which can commonly result in an intra-abdominal abscess. Abscesses such as these can have poor outcomes for patients if not recognized and treated with drainage of the source and appropriate antibiotic therapy.
